# Lactate, a Neglected Factor for Diabetes and Cancer Interaction

**DOI:** 10.1155/2016/6456018

**Published:** 2016-12-18

**Authors:** Yong Wu, Yunzhou Dong, Mohammad Atefi, Yanjun Liu, Yahya Elshimali, Jaydutt V. Vadgama

**Affiliations:** ^1^Division of Cancer Research and Training, Department of Internal Medicine, Charles R. Drew University of Medicine and Science, Los Angeles, CA 90059, USA; ^2^David Geffen UCLA School of Medicine and UCLA Jonsson Comprehensive Cancer Center, University of California, Los Angeles, CA 90095, USA; ^3^Vascular Biology Program, Boston Children's Hospital, Harvard Medical School, Boston, MA 02115, USA

## Abstract

Increasing body of evidence suggests that there exists a connection between diabetes and cancer. Nevertheless, to date, the potential reasons for this association are still poorly understood and currently there is no clinical evidence available to direct the proper management of patients presenting with these two diseases concomitantly. Both cancer and diabetes have been associated with abnormal lactate metabolism and high level of lactate production is the key biological property of these diseases. Conversely, high lactate contribute to a higher insulin resistant status and a more malignant phenotype of cancer cells, promoting diabetes and cancer development and progression. In view of associations between diabetes and cancers, the role of high lactate production in diabetes and cancer interaction should not be neglected. Here, we review the available evidence of lactate's role in different biological characteristics of diabetes and cancer and interactive relationship between them. Understanding the molecular mechanisms behind metabolic remodeling of diabetes- and cancer-related signaling would endow novel preventive and therapeutic approaches for diabetes and cancer treatment.

## 1. Introduction

Globally, diabetes mellitus (DM) and cancer are two of the most predominant diseases, with cancer the 2nd and diabetes the 12th primary cause of death [1, 2]. The connection between these two diseases was first hypothesized over 75 years ago. More and more evidence proposes that DM is related to an augmented risk of cancer [3] and the higher mortality in cancer patients [4, 5]. Actually, recent studies have suggested that type 2 diabetes (T2DM) is an independent risk factor for the progress of various types of cancer [6]. Although these two diseases share a number of common risk factors, the biological link between them is still not well known [6, 7], which poses a challenge for clinical management. While a thorough picture is yet to emerge, several mechanisms have been suggested to explain this relationship, for example, hyperglycemia itself [3], oxidative stress [8–11], treatment for diabetes, hormonal disorders, insulin resistance with secondary hyperinsulinemia [3], metabolic alterations underlying the diseases [12], insulin-increased bioactivity of IGF-I [13, 14], insulin's positive effect on estrogen bioavailability, the status of chronic inflammation, and obesity [7]. On the other hand, DM might also develop after tumor establishment in certain cancers that progress very rapidly, for example, pancreatic and liver cancers [15].

Lactate (2-hydroxypropanoic acid), formerly deemed a waste product of glycolysis, has drawn more and more attention as a crucial regulator of insulin resistance, DM, cancer development, maintenance, and metastasis. Over the last half century, substantial experiments revealed that lactate is both a powerful fuel and signaling molecule, and it is continuously being produced and circulated through the body [16]. Its presence in diabetes and cancer has been recognized, and recent studies suggest that suppressing it can be therapeutic, after 50 years of disavowal. Recently, cancer and DM have been associated with abnormal lactate metabolism. Lactate facilitates cancer cell intrinsic effects on metabolism and has extra noncancer cell autonomous effects which can induce tumorigenesis. In addition, lactate plays an important role in stimulating tumor inflammation and in promoting tumor angiogenesis by functioning as a signaling molecule [17]. Given that hyperlactacidemia is the most imperative biological feature of diabetes and cancer, it is reasonable to imagine that hyperlactacidemia might play an important role during diabetes and cancer interaction. Here, we review the available evidence of lactate's role in different biological characteristics of diabetes and cancer and interactive relationship between them. It appears that hyperlactacidemia may function as an interaction hub between diabetes and cancer and contribute to a higher insulin resistant status and a more malignant phenotype of cancer cells.

## 2. Lactate Production and Metabolism

Lactate, a 3-carbon hydroxycarboxylic acid, is produced in the cytoplasm by the glycolysis pathway under anaerobic conditions, via the reduction of an intermediate metabolite pyruvate, with the simultaneous oxidation of NADH to NAD+. This reaction is catalyzed by lactate dehydrogenase (LDH) [18]. LDH is composed of four subunits of two distinct types (H and M), with each subunit type under distinct genetic control leading to five diverse isozymes including LDH-1 (H4), LDH-2 (H3M1), LDH-3 (H2M2), LDH-4 (H1M3), and LDH-5 (M4) [19]. Under aerobic conditions and in the presence of the enzyme pyruvate dehydrogenase (PDH), pyruvate is converted into acetyl CoA, subsequently entering the tricarboxylic acid (TCA) cycle or Kreb's cycle.

**Figure 1:**
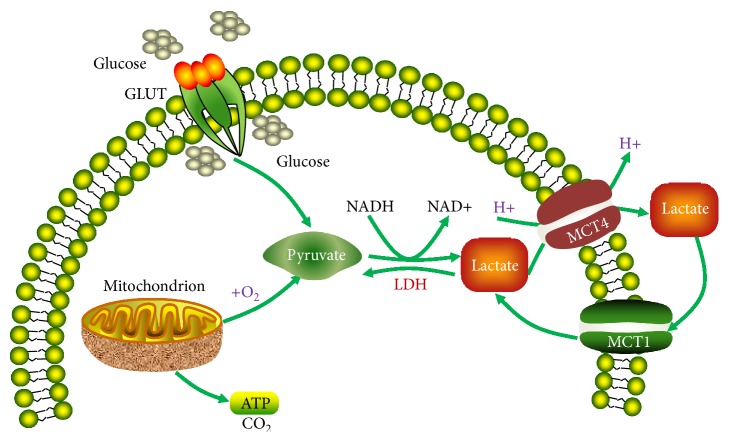
Lactate production and shuttling pathways. GLUT, glucose transporter; LDH, lactate dehydrogenase; MCT, monocarboxylate transporter.

The normal plasma concentration of lactate is 0.3–1.3 mM. In plasma, lactate is buffered by NaHCO_3_. Lactate may have two stereoisomers, namely, d-lactate and l-lactate. In humans, lactate exists predominantly in the levorotatory isoform. Most tissues in the human body produce lactate, but the majority of production is found in muscles [18]. Lactate is transported across the plasma membrane with the aid of the monocarboxylate transporters (MCTs), which facilitates the proton-linked transport of monocarboxylates, for example, L-lactate, pyruvate, and the ketone bodies [20, 21]. So far four isoforms, MCT1–4, have been functionally substantiated to implement this function in mammals, each with different substrate and inhibitor affinities [20, 21] (Figure 1).

Plasma concentrations of lactate represent an equilibrium between its production and metabolism. Lactate can be metabolized by various cells and tissues, for example, liver, germ cells, and neurons, converting to pyruvate via LDH and subsequently to glycogen or carbon dioxide [22]. Under normally physiological conditions, lactate is cleared by the livers and kidneys [23, 24]. At present, lactate is also considered as a regulator of energy homeostasis [16, 25, 26]. At a generalized level, lactate can be carried to the liver and reconverted into glucose through the Cori cycle, serving as an energy source [27].

## 3. Lactate Production Increases in Diabetes

Fasting plasma lactate level is increased in patients with DM including T1DM and T2DM versus nondiabetic persons [28–36]. Diabetic patients with obesity exhibit higher fasting plasma lactate levels than nondiabetic individuals with obesity [37, 38]. Barnett et al. proposed that diabetes-associated hyperlactatemia might be an early change in the time course of the disease [39]. Recently, Berhane et al. [40] demonstrated that lactate production progressively rises during hyperinsulinemic euglycemic clamp study, a condition of hyperinsulinemia similar to the early stages in the development of T2DM. Intriguingly, similar previous studies also report elevated lactate concentrations during the early stages of diabetes, prediabetes, and the hyperinsulinemia condition. In addition, Brouwers et al. [41] reported increased lactate levels in patients with poorly controlled T1DM and glycogenic hepatopathy, implying that enhanced plasma lactate concentrations are part of the clinical spectrum of these diseases. Furthermore, lactate has also been revealed to predict diabetes occurrence in the future [42, 43].

The mechanisms underlying diabetes-associated hyperlactatemia include serious changes in the intracellular glucose metabolism in insulin-sensitive tissues, for example, diminished glycogen synthesis, compromised glucose oxidative metabolism, and increased whole-body rate of nonoxidative glycolysis [28, 31, 44]. Importantly, when compared with controls, nonoxidative glycolysis rate retains higher in T2DM patients during hyperglycemic [31, 44, 45] and hyperinsulinemic [31, 44] status. In addition, the postprandially nonoxidative glycolysis is elevated in these patients relative to healthy controls and blood lactate level rises under this condition [36]. Insulin resistance plays a vital role in the pathogenesis of T2DM [46] and can be used as an early marker for the disease [40]. Under the insulin resistant condition, high levels of insulin promote glycolysis through activating two rate limiting enzymes, namely, phosphofructokinase and pyruvate dehydrogenase [47]. Thus, patients with insulin resistance/diabetes exhibit augmented activity of glycolysis [31, 48]. The elevated glycolysis results in enhanced formation of NADH and pyruvate and reduced NAD+ levels. Pyruvate is converted into lactate by LDH accompanied by NAD+ generation from NADH in a redox reaction. This reaction may be accentuated in insulin resistance since hyperinsulinemia induces enhanced glycolysis.

## 4. Contribution of Lactate to Insulin Resistance/Diabetes

As an imperative cellular metabolite in the glycolytic pathway, lactate might reflect the cellular metabolism status. Some studies suggest that augmented lactate levels in obesity, which might play a significant role in glucose transport and metabolism, profoundly influence insulin sensitivity [49]. Its high plasma level might be an early indication of the beginning of insulin resistance and can be utilized to identify a state of insulin resistance [40]. In addition, in HIV-infected patients treated with nucleoside reverse transcriptase inhibitors, both resting and postexercise levels of lactate are associated with insulin resistance in skeletal muscle [50]. Lactate alone or combined with other insulin secretagogues, for example, ketone bodies, stimulates insulin release in INS-1 cells and isolated pancreatic islets [51], indicating that increased plasma lactate promotes insulin secretion and pancreatic response to insulin secretagogues. Thus, these results suggest that lactate not only enhances insulin secretion from *β*-cells but also improves the responsiveness of these cells to insulin [51]. These data may explain that the transiently elevated lactate obtained during physical exercises and aerobic/anaerobic training improves DM symptoms. Instead, lactate concentrations are chronically increased in diabetic patients with obesity [52]. The chronical hyperlactatemia maintained by the enhanced lactate formation from adipocytes in obese individuals [53] is found preceding diabetes onset [52] and might participate in this pathologic process. Together, these data indicate that chronical hyperlactatemia might indicate the early stages of insulin resistance and contributes to the onset of diabetes. Actually, some epidemiologic studies suggest that high lactate levels might predict the occurrence of diabetes [42, 43]. Crawford et al. [43] in their cross-sectional study among white elderly people with severe carotid atheromatosis reveal a relationship between plasma lactate levels and prevailing T2DM; nonetheless no association is detected among African Americans.

While the molecular mechanisms underlying lactate-induced insulin resistance/diabetes are yet uncertain, it has been proposed that inhibition of the ability to oxidize glucose, the repression of glucose transport, and insulin-stimulated glycolysis, as well as reduced insulin-induced glucose uptake is implicated in this phenomenon. Furthermore, it has been suggested that lactate-induced insulin resistance is related to compromised insulin signaling and reduced insulin-triggered glucose transport in skeletal muscle [54].

## 5. Lactate Production Increases in Cancer

A common feature of primary and metastatic cancers is increase in glycolysis rate, leading to augmented glucose uptake and lactate formation, even under normal oxygen conditions. This is also known as aerobic glycolysis or the “Warburg effect” [55], a metabolic hallmark of cancer. It was first described in the 1920s by Warburg and he hypothesized that cancer is caused by compromised mitochondrial metabolism. While this hypothesis has been proven wrong, the experimental observations of elevated glycolysis in cancers even under normoxic conditions have been repetitively substantiated [56]. Unlike anaerobic glycolysis that stimulates energy generation under hypoxia, the Warburg effect provides a proliferative advantage via converting carbohydrate fluxes from energy generation to biosynthetic processes. To meet cancer cell proliferation requirements, the glycolytic switch is related to increased glucose consumption and lactate accumulation [57]. It is shocking that the lactate levels determined in human cancers, for example, cervix cancer, can range from 4 mM to 40 mM [58], while the physiological levels of lactate in normal tissues are 1.8–2 mM [59].

The molecular mechanisms underlying upregulation of glycolysis in cancer are not well delineated. It is generally assumed that this phenomenon results from defective cellular respiration, oncogenic changes, and overexpression of metabolite transporters and glycolytic enzymes, for example, glucose transporters and hexokinases, which are the crucial regulatory molecules for glycolytic flux [60]. The oncogenes and tumor suppressor genes implicated in the metabolic alteration from oxidative phosphorylation to an increased glycolysis of cancer cells include hypoxia-inducible factor-1*α* (HIF-1*α*) [60, 61], epidermal growth factor (EGF), phosphoinositol 3-kinase (PI3-K), myc, nuclear Factor Kappa Beta, protein kinase B (PKB), insulin-like growth factor I, mTOR, Kirsten rat sarcoma viral oncogene homolog (KRAS), and 5′ adenosine monophosphate-activated protein kinase (AMPK). The majority of these oncogenes stimulate genes encoding proteins that regulate glycolysis and glutaminolysis [55].

Among the aforementioned oncogenes, the transcription factor HIF-1*α* is the most important controller of the glycolytic response and cellular adaptation [62]. Expression of HIF-1*α*-regulated genes results in an increased glycolytic flux in cancer cells in an oxygen-independent manner. The targets of HIF-1 include hexokinase II [63], angiogenic growth factors (e.g., VEGF), haematopoietic factors (e.g., erythropoietin and transferrin) [64], and membrane transporters including glucose transporter-1 (GLUT-1) and monocarboxylate transporter-4 (MCT-4). These membrane transporters contribute to both sufficient glucose transport into the cell and release of amassed lactate out of the cell. HIF-1*α* activates pyruvate dehydrogenase kinase 1 (PDK-1) and subsequently inactivates the pyruvate dehydrogenase complex (PDC), leading to reduced flux into oxidative phosphorylation [55]. In addition, the activated HIF-1*α* is related to constitutively high rate of glucose consumption. Furthermore, hypoxia-reoxygenation injury in cancers may stabilize HIF-1*α* [65], indicating that its constitutive upregulation may be caused by the cyclic oxic-hypoxic cycles which happen in premalignant cancers.

In addition to glycolysis, glutaminolysis is another primary pathway for energy generation and cause increased lactate formation in cancer cells. Moreover, glutaminolysis facilitates macromolecule synthesis in proliferating tumor cells [61]. The tumor-specific isoform of pyruvate kinase (PK) M2 (PKM2) offers an additional source of lactate by converting phosphoenolpyruvate (PEP) into pyruvate. Nevertheless, PEP may promote the production of pyruvate independent of PKM2 activity through serving as a phosphodonor for phosphoglycerate mutase 1 (PGAM1) [66].

## 6. Lactate Facilitates Cancer Development

High concentrations of lactate have been linked to unfavoured clinical outcome in some human cancers [57]. Augmented intratumoral lactate levels are related to elevated incidence of metastasis in cervical, breast, head, and neck cancers [58, 67, 68]. Due to lactate concentrations conversely correlated with overall and disease-free patient survival, tumor lactate generation, serum lactate, and LDH levels have long been recognized as prognostic biomarkers of patients with various types of epithelial cancers [55, 69–79]. Increased lactate alters microenvironment, fuels cancer cells, and results in acidosis, inflammation, angiogenesis, immunosuppression, and radio-resistance [80–83]. In the next paragraphs, we review these biological actions of increased lactate in cancer development and progress by describing the main evidences.

Substantial studies have demonstrated that cancer cells can uptake lactate and use it for energetic production and amino acid formation. Accumulative evidence demonstrates that lactate is a fuel for the oxidative metabolism in oxygenated cancer cells [68, 84–87] and a signaling mediator in cancer and endothelial cells (ECs) [88–90]. Recently, Bonuccelli et al. [68] reveal that ketones and lactate fuel tumor growth and metastasis, which might illuminate why diabetic patients have an augmented cancer incidence and poor prognosis, because of elevated ketone/lactate production. In vitro studies suggest that cervical cancer SiHa cells and breast cancer MDA-MB-231 cells uptake lactate in a pH-dependent manner [84, 91]. Due to lack of sufficient oxygenation or an effective vascular network in the microenvironment, cancer uptake and exploitation of lactate is dependent on oxygen concentrations, lactate levels, amount of healthy mitochondria, and suitable MCT expression [92, 93]. Owing to the significant metastasis-promoting characteristics of lactate, one can reason that it is unwise to use lactate-containing intravenous injection solutions, for example, lactated Ringer's or Hartmann's solution in cancer patients [68].

The tumor microenvironment (TME) refers to a sophisticated network of extracellular matrix molecules, soluble factors, adipocytes, and stromal cells including tumor endothelial cells (TECs), tumor-associated fibroblasts (TAFs), and macrophages. Among the soluble factors in TME, large amounts of lactate are important due to its effects on tumor and stromal cells [18]. In addition, it decreases extracellular pH to 6.0–6.5 [94–96]. Actually, lactic acidosis frequently contributes to death in patients with some types of metastatic cancer, for example, metastatic breast cancer [97–113]. The acidic TME causes pain in cancer patients [114] and results in metastasis of some tumors [115]. Moreover, acidosis per se may be mutagenic [116], probably via suppression of DNA repair [95] and may result in spontaneous transformation of diploid fibroblasts [117]. Under some circumstances, low pH induces in vitro invasion [118] and in vivo metastasis [119], possibly via the metalloproteinases/cathepsins, which stimulate the degradation of the extracellular matrix and basement membranes [120, 121]. Lactic acidosis results in overexpression of matrix metalloproteinase-9 (MMP-9) [122], VEGF-A [123, 124], transforming growth factor-*β*2 (TGF-*β*2) [125] and IL-8 [126–128] in various cancer cells, rendering the TME even more complicated. Pavlides et al. [129] suggest that cancer cells stimulate aerobic glycolysis in CAFs. CAFs render tumor survival and a higher proliferative capacity by a number of factors including secreting lactate and pyruvate and alterations in cell metabolism. Accordingly, cancer cells may become accustomed to rapid alterations in the TME via reprograming stromal cells and via the metabolic interchange between oxidative and glycolytic cells [129, 130].

Within the tumor, TAFs exhibit a different lactate metabolic pathway than the cancer cells. TAFs mainly contain low levels of glucose importer GLUT1, lactate dehydrogenase-B and pyruvate dehydrogenase, while cancer cells contain high GLUT1, lactate dehydrogenase-A, pyruvate dehydrogenase kinase and hypoxia inducible factor-1*α*. Within cancer cells, the imported glucose is metabolized to pyruvate, while pyruvate dehydrogenase is inactive due to its phosphorylation by pyruvate dehydrogenase kinase phosphorylates. Therefore, LDH-5 (made of LDHA subunits) in an anaerobic manner converts pyruvate to lactate which is exported out of the cell. On the other hand, TAFs import the lactate and by their LDH-1 (containing LDHB subunits) activity convert it back to pyruvate which is funneled to aerobic pathways of mitochondria via the activity of pyruvate dehydrogenase. It seems that these two lactate metabolic pathways in cancer cells and TAFs work in a complementary manner as cancer cells generate high levels of lactate and acidify the microenvironment while TAF consume the lactate in an aerobic manner and decrease the acidity of the microenvironment [131, 132].

The angiogenesis process supports the new blood vessel development and plays an important role in restoring perfusion, oxygenation, and nutrient supply. Lactate is an imperative contributor to wound healing and angiogenesis [133–135]. Lactate itself induces cell migration [134], vascular morphogenesis [136], circulating vascular progenitor cell recruitment [137], and tube formation and promotes angiogenesis by activating the VEGF/VEGFR2 pathway [136, 138] and stimulating endothelial cells via MCT1, which induces the phosphorylation and degradation of I*κ*B*α*, triggering the NF-kB/IL-8 (CXCL8) signaling pathway [90]. Lactate-stimulated angiogenesis depends on lactate oxidation by LDH-1, exploiting the enzymatic reaction products, for example, pyruvate and NADH, and lactate transporters [136, 137]. The enhancing production of pyruvate from lactate oxidation activates NF-*κ*B and HIF-1, leading to overexpression of some growth factors required for angiogenesis, including VEGF, basic fibroblast growth factor (bFGF), and stromal cell-derived factor-1 (SDF-1) [139, 140]. In addition, Vegran et al. [90] demonstrate that lactate-stimulated NF-*κ*B activation in ECs is associated with IL-8-mediated autocrine angiogenesis and that this pathway promotes EC migration and tube formation in vitro, as well as lactate-triggered tumor angiogenesis in vivo.

Endothelial cells of tumor vasculature import high levels of glucose (high GLUT1 levels). However, since they contain high LDH1 and low HIF-1*α* and lowLDH5, similar to TAFs, they show an aerobic metabolism. Meanwhile due to low expression of lactate transporters, endothelial cells perhaps do not import much of the lactate in the tumor. Hence, it seems the main role of endothelial cells is to respond to the tumor microenvironment by generating new vessels to support the cancer cells and other tumor associated cells. However, they may not participate in uptake and consumption of lactate within the tumor [132, 141].

One main reason for cancer development is that the immune system loses its ability to effectively eradicate aberrant cells. High levels of lactate have a harmful effect on the tumor infiltrating immune cells. Clinical evidence indicates that lactate restricts immune cell infiltration in renal cell carcinoma (RCC) and damages the metabolism and cytolytic functions of T cells in the TME [80, 142]. Lactate hinders proliferation and cytokine release of human cytotoxic T lymphocytes (CTLs) by 95% and their cytotoxic activity by 50%. Lactate released from melanoma cells impedes TAA-induced IFN-*γ* generation by specific CTLs in melanoma spheroid cocultures [143]. In addition, other studies substantiated that high levels of lactate suppresses TCR-stimulated cytokine release (IFN-*γ*, TNF-*α*, and IL-2) and prompts partial damage of lytic granules exocytosis in CTLs by selectively downregulating the MAPKs p38 and JNK/c-Jun signaling pathways [81]. Moreover, tumor-derived lactate enhances arginase-1 (ARG1) expression in tumor-associated macrophages (TAMs), hindering T-cell activity and proliferation [144], inhibiting antitumor immune responses and promoting tumor growth [145, 146]. Lately, Colegio et al. [145] demonstrated that, under normoxic conditions, lactate stabilizes HIF-1*α*, resulting in ARG1 and VEGF gene expression in macrophages. Furthermore, tumor-derived lactate changes monocytes' function hinders their differentiation to DCs and inhibits the cytokine production from differentiated DCs and suppresses the activity of NK cells, thus contributing to immune suppression within tumors [82, 147, 148].

Some studies on experimental tumors, including about 1,000 xenografts of individual human head and neck squamous cell carcinoma, indicate that lactate levels are positively correlated with radio-resistance [149]. The mechanisms behind this correlation reside in, at least partially, the antioxidant characteristics of lactate [150]. Anticancer treatments, for example, ionizing radiation and a number of chemotherapeutic drugs, work through inducing overproduction of reactive oxygen species (ROS) in targeted cancer cells, which causes DNA/RNA damage, genomic instability, and lipid peroxidation. Hence, an accretion of lactate may promote resistance to radiation and lead to chemoresistance [151]. Wagner et al. reveal that lactate can modulate cellular DNA damage repair processes in the uterine cervix, leading to the resistance of cervical cancer cells to anticancer therapy [152]. Since animals receiving chemotherapy or radiotherapy exhibit a reduction in lactate [153], checking this metabolite in human cancers might be used to predict therapeutic responses. Accordingly, a recent study [154] proposes that lactate can be used as a quantitative biomarker of acute radiation response.

Finally, lactate is a mediator of inflammation [155, 156] and might be used as a biomarker of inflammatory processes [157]. Lactate and inflammation stimulate each other in a malicious cycle [83]. It promotes IL-4/IL-13 production [158] and stimulates the IL-23/IL17 pathway [18]. Lactate promotes IL-23p19 expression in tumor infiltrating immune cells by stimulating toll-like receptor. In addition, it stimulates splenocytes to secrete IL-17 in an IL-23-dependent manner. These effects stimulate local inflammatory responses, favoring the incidence and development of tumors [159]. In addition, lactate benefits the growth of inflammation-associated colorectal tumor by promoting PGE2 synthesis and gluconeogenesis in monocytes [160]. Together, these studies suggest that lactate plays a significant proinflammatory role in tumor development.

It is believed that in diabetic patients, the adipose tissue plays a major role in induction of metabolic syndrome. In these patients there is an underlying chronic inflammation in adipose tissue and a general increase in levels of cytokines such as TNF-*α*, IL-1, and IL-6 [161]. While these released factors play important roles in cancer biology, there is evidence that points to their possible reciprocal roles in the lactate level. For instance, TNF*α* can induce LDHA and lactate production in a short period of time [162], while lactate induces release of TNF-*α* and IL-6 in some cells [163]. In a study on rats, chronic infusion of IL-1*α* induced hyperlactacidemia [164] and in another study on rat ovaria cells, IL-1*β* enhanced glucose uptake and induced aerobic glycolysis [165]. Moreover, it has been shown that high levels of IL-6 correlated with high levels of lactate and can result in poor prognosis of patients with metastatic melanoma [166]. These findings indicate that the release cytokines may play roles in both cancer and metabolic syndrome and may be the connecting points between developments of both diseases.

## 7. Concluding Remarks and Future Perspectives

**Figure 2:**
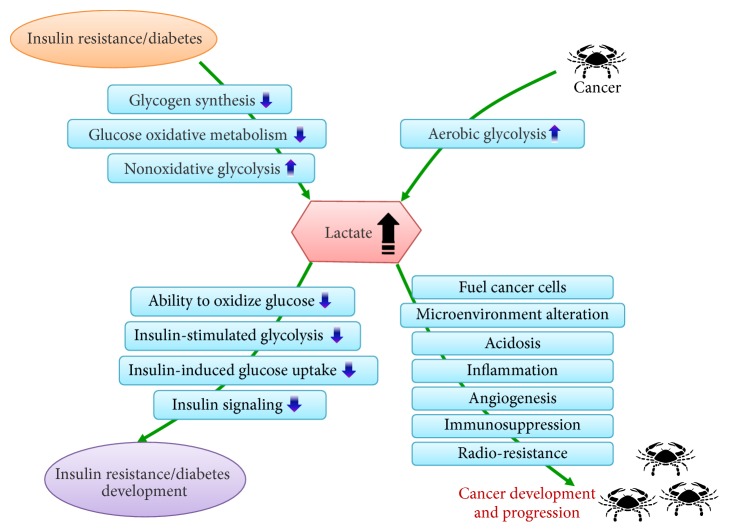
Illustration of lactate as an interaction hub between diabetes and cancer.

Accumulative evidence indicates a high incidence and mortality for a variety of malignancies in patients with diabetes. Diabetes and its risk factors are associated with cancer and they have an intricate and reciprocally reinforcing relationship. Nevertheless, the underlying mechanisms are poorly understood and currently there is no clinical evidence available to direct the proper management of patients presenting with these two diseases concomitantly. Diabetes and cancer interact with each other in a vicious cycle, where lactate plays a pivotal role in this mutual interaction. Insulin resistance/diabetes and cancer conditions produce high levels of lactate and conversely high lactate promotes diabetes and cancer development and progression (Figure 2).

In diabetes, hyperlactacidemia is perhaps due to the high levels of insulin which induces the activity of two glycolytic enzymes phosphofructokinase and pyruvate dehydrogenase [47]. However, glycolytic switch in cancer is due to the increased activity of glycolytic enzymes, for example, glucose transporters and hexokinases [60], which have been attributed to signaling pathways such as HIF-1*α* [60, 61], EGF, phosphoinositol 3-kinase (PI3-K), myc, NF-kB, PKB, IGF-I, mTOR, KRAS, and AMPK. Among these, HIF-1 signaling seems to be very important as it induces hexokinase II [63], GLUT-1 and MCT4, and pyruvate dehydrogenase kinase 1 (PDK-1), and therefore inactivates the pyruvate dehydrogenase, leading to reduced flux into oxidative phosphorylation [55]. In both diabetes and cancer lactate can induce inflammation through IL-4/IL-13 production [158] and IL-23/IL17 pathway [18]. However, in cancer the effect of lactate is more profound and can alter microenvironment, fuels cancer cells, and results in acidosis, inflammation, angiogenesis, and immunosuppression [80–83].

In this review, we deliberated the mechanisms underlying high lactate induced by diabetes and cancer, as well as the effects of high level of lactate production, and the key property of diabetes and cancer, on diabetes development and different cancer biological behaviors. Besides supplying abundant nutrition for tumor growth, increased lactate level might also activate various signaling pathways, which play imperative roles in cancer development and progression. Existing evidence demonstrates that some diabetes treatments might have significant therapeutic implications in cancer patients and that MCT/lactate transport inhibitors are employed therapeutically to repress cancer metastasis. Understanding the molecular mechanisms behind metabolic remodeling of diabetes- and cancer-related signaling would endow novel preventive and therapeutic approaches for diabetes and cancer treatment. Importantly, combined management of diabetes and cancer probably leads to better improvement in mortality versus treating them individually. Accordingly, more interdisciplinary approaches are required to reveal the mechanisms underlying the links between these two diseases and, eventually, ameliorate clinical outcomes.
